# Phylogeny and palaeoecology of *Polyommatus* blue butterflies show Beringia was a climate-regulated gateway to the New World

**DOI:** 10.1098/rspb.2010.2213

**Published:** 2011-01-26

**Authors:** Roger Vila, Charles D. Bell, Richard Macniven, Benjamin Goldman-Huertas, Richard H. Ree, Charles R. Marshall, Zsolt Bálint, Kurt Johnson, Dubi Benyamini, Naomi E. Pierce

**Affiliations:** 1 Museum of Comparative Zoology, Harvard University, 26 Oxford Street, Cambridge, MA 02138, USA; 2 ICREA and Institute of Evolutionary Biology (CSIC-UPF), Passeig Marítim de la Barceloneta 37–49, Barcelona 08003, Spain; 3 Department of Biological Sciences, University of New Orleans, 2000 Lakeshore Drive, New Orleans, LA 70148, USA; 4 Biogen Idec, 14 Cambridge Center, Cambridge, MA 02142, USA; 5 Department of Ecology and Evolution, The University of Arizona, 424 Biosciences West, Tucson, AZ 85721, USA; 6 Department of Botany, Field Museum of Natural History, 1400 South Lake Shore Drive, Chicago, IL 60605, USA; 7 University of California Museum of Paleontology, and Department of Integrative Biology, University of California, Berkeley, 1101 Valley Life Sciences Building, Berkeley, CA 02138, USA; 8 Department of Zoology, Hungarian Natural History Museum H-1088, Budapest, Baross utca 13, Hungary; 9 Florida State Collection of Arthropods/McGuire Center, University of Florida Cultural Plaza, Hull Road, Gainesville, FL 32611, USA; 10 91 Levona Street, Bet Arye, 71947, Israel

**Keywords:** Beringia, biogeography, climate change, Lycaenidae, Nabokov, phylogeny

## Abstract

Transcontinental dispersals by organisms usually represent improbable events that constitute a major challenge for biogeographers. By integrating molecular phylogeny, historical biogeography and palaeoecology, we test a bold hypothesis proposed by Vladimir Nabokov regarding the origin of Neotropical *Polyommatus* blue butterflies, and show that Beringia has served as a biological corridor for the dispersal of these insects from Asia into the New World. We present a novel method to estimate ancestral temperature tolerances using distribution range limits of extant organisms, and find that climatic conditions in Beringia acted as a decisive filter in determining which taxa crossed into the New World during five separate invasions over the past 11 Myr. Our results reveal a marked effect of the Miocene–Pleistocene global cooling, and demonstrate that palaeoclimatic conditions left a strong signal on the ecology of present-day taxa in the New World. The phylogenetic conservatism in thermal tolerances that we have identified may permit the reconstruction of the palaeoecology of ancestral organisms, especially mobile taxa that can easily escape from hostile environments rather than adapt to them.

## 1. Introduction

Butterflies have been avidly studied for centuries, and our knowledge of their natural history, taxonomy and distribution is arguably the best better than for any other invertebrates. However, their evolutionary relationships and biogeographic history are far from understood. This is especially true for the Polyommatini, commonly known as the ‘blues’, which is among the largest and most systematically challenging tribes within the family Lycaenidae (the blues, coppers and hairstreaks). Since Eliot's classification of the Lycaenidae in 1973, no comprehensive revision of this tribe has been attempted, and even Eliot admitted ‘complete failure’ in his efforts to subdivide it into natural groups, simply organizing it into 30 sections [1]. With more than 400 species, the cosmopolitan *Polyommatus* section (equivalent to ‘Plebejinae’ in older classifications) is the most diverse of these. These butterflies are specialists of seasonal habitats with extreme dry or cold periods, and can be found from deserts to grasslands to Alpine and Arctic tundra. While their centre of diversity is clearly in the Palaearctic, many species occur in the New World, especially in the Neotropics.

**Figure 1.**
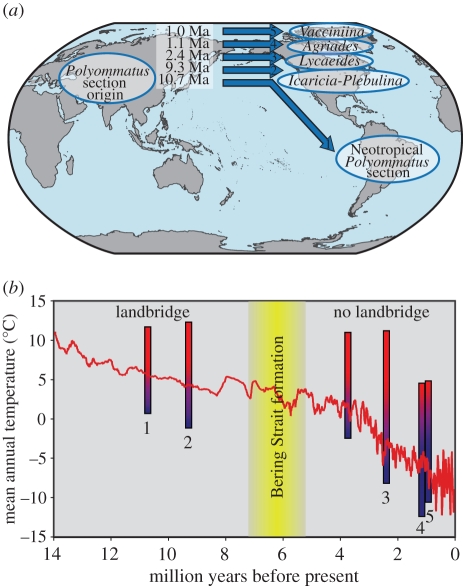
(*a*) Nabokov's hypothesis for the colonization of the New World by butterflies in the section *Polyommatus* (Lepidoptera: Lycaenidae). Results of this study fully support his proposal of five dispersals of *Polyommatus* blues across Beringia over the last 11 Myr, in the following order. The first stock (1: *ca* 10.7 Ma) expanded southwards to the Neotropics; the other four colonization events produced the *Icaricia*–*Plebulina* clade (2: *ca* 9.3 Ma) and the New World *Lycaeides* (3: *ca* 2.4 Ma), *Agriades* (4: *ca* 1.1 Ma) and *Vacciniina* (5: *ca* 1.0 Ma). (*b*) Estimated temperature tolerance ranges of the five ancestors that crossed Beringia plotted against the time of each colonization event. Ancestral temperatures at Beringia versus time (red line) are also shown. Note that the temperature tolerance ranges of colonizing ancestors decreased significantly with time (*p* < 0.01), closely following the Miocene–Pleistocene climate change trend, and that the coldest temperatures tolerated by each of the ancestors predict their capacity to survive and cross Beringia at the time of the colonization events.

The radiation of *Polyommatus* blues in the New World was first appreciated by the famous writer Vladimir Nabokov when he was working as curator in the Museum of Comparative Zoology at Harvard in the early 1940s. Although sometimes described as an amateur [2,3], Nabokov was a serious taxonomist who made important contributions to the systematics of the *Polyommatus* section and revised many of the New World taxa, particularly in the genus *Lycaeides* [4–7]. In his most significant paper, published in 1945 [8], Nabokov drastically rearranged the Neotropical taxa, describing seven new genera [2,3]. Importantly, Nabokov laid out a detailed phylogeographic hypothesis for the New World *Polyommatus* blues [8] (figure 1*a*). Nabokov described how ‘a modern taxonomist straddling a Wellsian time machine with the purpose of exploring the Cenozoic era’ would encounter the following series of events in the evolution of these butterflies. (i) From Asian ancestors, a first colonization event of the New World across the Bering Strait, followed by dispersal southwards to South America. This first stock would produce the current Neotropical taxa, but would subsequently vanish almost completely from North America. (ii) A second crossing of the Bering Strait made by the ancestors of the *Icaricia*–*Plebulina* clade. And finally, more recently, the dispersal of (iii) *Lycaeides*, (iv) *Agriades* and (v) *Vacciniina* (explicitly in that order) from Asia to North America following the same route.

Several alternative hypotheses could explain the colonization of the New World by *Polyommatus* blues. The first Nabokov mentions but discards: transoceanic landbridges in other parts of the world. Nabokov was writing in 1945, before the concept of continental drift had reached general acceptance. Taking continental drift into account, the pattern of evolution of *Polyommatus* blues could conceivably be explained by the break-up of western Gondwana. This hypothesis has sometimes been proposed for the origin for the Lycaenidae [1], but requires that these butterflies evolved much earlier than commonly believed [9]. More recently, Bálint & Johnson [10], extending morphological studies to the recently discovered taxa, proposed an alternative hypothesis in which Neotropical taxa have multiple origins and are closely related to Old World taxa, some even belonging to other sections.

Here we present the first comprehensive molecular phylogeny of the Polyommatini tribe and *Polyommatus* section, and investigate the biogeographic history of the New World *Polyommatus*, taking into account phylogenetic relationships as well as inferred palaeoecology. We explicitly investigate the use of Beringia as a corridor for dispersal by using distribution ranges of current taxa to estimate ancestral temperature tolerances that would have affected the dispersal abilities of these insects over the past 11 Myr.

## 2. Material and methods

Methods are described in greater detail with full references in the electronic supplementary material.

### (a) Taxon sampling

The phylogenetic analysis at the tribal level included 11 ingroup taxa (four from the Old World and seven from the New World), plus 39 outgroup taxa, representing a total of 28 sections. For the section-level phylogeny, we used 73 representatives of the *Polyommatus* section (20 Old World and 53 New World taxa) that included at least one representative for each New World genus/subgenus, and all the Old World taxa that have been hypothesized as possibly related to them. Representatives of the *Everes* and *Leptotes* sections were used as outgroups.

### (b) Sequencing, alignment and phylogenetic analyses

Total genomic DNA was extracted from the specimens, and fragments from two mitochondrial markers—*Cytochrome Oxidase I* (*COI*)–(*leu-tRNA*)–*Cytochrome Oxidase II* (*COII*)—and six nuclear markers—*Elongation Factor-1 alpha* (*EF-1*α**), *28S ribosome unit* (*28S*), *Histone H3* (*H3*), *wingless* (*wg*), *carbamoyl-phosphate synthetase 2/aspartate transcarbamylase/dihydroorotase* (*CAD*) and *internal transcribed spacer 2* (*ITS-2*) [11]—were amplified by polymerase chain reaction and sequenced. All sequences were submitted to GenBank under accession numbers GQ128446–GQ129111. Alignments were unambiguous for protein-coding genes. ClustalX (v. 1.83.1) [12] was used to align *28S* and *ITS-2*, and, in the case of the latter, ambiguous regions were excluded from the analyses. Phylogenetic analyses were conducted using maximum parsimony (MP; PAUP* v. 4.0b10 [13]), maximum likelihood (ML; GARLI v. 0.951 [14] and RAxML v. 2.0 [15]) and Bayesian inference (BI; MPI-enabled version of MrBayes v. 3.1.2 [16]) for each marker, each genome and for all markers combined. The program PORN* [17] was used to determine the best-fitted substitution model for each data partition in ML and BI analyses. The Akaike information criterion was used to evaluate the fit of competing models. In all cases the GTR + *Γ* model was selected as the most appropriate. Each BI analysis consisted of six independent 5-million-generation runs, with four chains (one cold and three hot) each. Non-parametric bootstrap values were used to estimate the support of tree branches recovered by MP and ML.

### (c) Dating main phylogenetic events

The topology of the ML phylogram recovered from the combined analysis with GARLI was used to date main phylogenetic events. The likelihood ratio test found a significant deviation from substitution rate consistency (*p* < 0.001) across different branches on the ML topology. We used two different methods: a strict molecular clock and penalized likelihood. The software r8s [18] was used to perform the rate-smoothing procedures. For *COI*, a slow rate of 6.5 × 10^−9^, an intermediate substitution rate of 7.5 × 10^−9^ and a fast substitution rate of 9.5 × 10^−9^ substitutions site^−1^ yr^−1^ were used [19]. For *COI* + *leu-tRNA* + *COII*, a substitution rate of 11.5 × 10^−9^ substitutions site^−1^ yr^−1^ was used [20]. Throughout the paper, the mean of ages obtained using eight different combinations of methods and rates is used as the best possible age estimate.

### (d) Ancestral area reconstruction

We used the software program DIVA v. 1.1 [21] and an improved version of Lagrange [22] to estimate ancestral areas and dispersals within the ingroup. We coded areas as Africa, Australia, Central America–Caribbean, East Nearctic, East Palaearctic, Northern South America, Oriental, Southern South America, West Nearctic and West Palaearctic. The biogeographic model permitted bidirectional dispersals between neighbouring regions that do not imply long transoceanic dispersals, plus north Atlantic and north Pacific transoceanic dispersals. The terminals were coded based on the genus distribution, except in the case of genera with more than one representative in the analysis, which were coded based on the species distribution range of the terminals. In the case of species with representatives in both New and Old World (*Agriades glandon*, *Lycaeides idas* and *Vacciniina optilete*), the taxa and distribution ranges in each part of the world were treated independently. Analyses were performed on the BI and the GARLI ML trees estimated from the combined dataset or the *COI* dataset, with identical results.

### (e) Ancestral character state reconstruction

Hostplant family was coded as a multi-state unordered character based on published and personal observations. Mesquite v. 2.6 [23] with MP character optimization was used on both Bayesian and GARLI ML trees estimated from the 78-taxa combined dataset. Present-day mean annual temperatures of coldest and warmest locations were obtained based on a survey that covered each taxon's global distribution range and focused on their latitudinally and altitudinally most extreme localities. Temperatures were coded as ordered continuous characters based on WorldClim v. 1.4 [24], and ancestral states were reconstructed with BayesTraits beta v. 1.1 [25] on the GARLI ML phylogram estimated from the 78-taxa combined dataset. The analysis performed is a Bayesian implementation of the comparative method used by the software Continuous [26]. We first tested the two available models, covariance of the two characters, and the use of lambda, delta and kappa parameters. We used 5 million iterations, a burn-in of 5000 and a sample period of 100 in each case. The significantly best model and set of parameters was used to reconstruct the ancestral character states of the nodes that involved the crossing of Beringia from the Old to the New World, according to Lagrange ancestral area reconstruction. The results were plotted against the mean age estimation for each of these nodes. The palaeoclimatic curve at Beringia was obtained using Zachos *et al*.'s [27] global *δ*^18^O curve, which was smoothed using a 50-point running mean and calibrated according to the present-day mean annual temperature (−9°C, according to WorldClim data for relatively warm localities in the Beringia region), and ancestral mean annual temperatures at 9 Ma (4°C) and 14 Ma (11°C) [28].

## 3. Results

**Figure 2.**
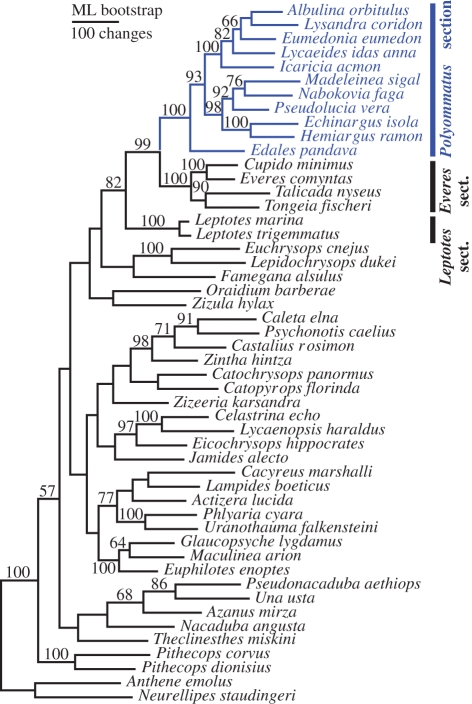
Phylogeny of the tribe Polyommatini (Lepidoptera: Lycaenidae). ML phylogram for 50 taxa representing 28 of the 29 sections (*sensu* [1], excluding *Cupidopsis*) inferred from 4939 bp of two mitochondrial and four nuclear markers (*COI*–(*leu-tRNA*)–*COII*, *EF-1*α**, *28S*, *H3* and *wg*). The *Polyommatus* section is monophyletic and sister to the *Everes* section, with both sister to the *Leptotes* section. Numbers above branches indicate bootstrap support greater than 50%. The analysis was done under the GTR + *Γ* model for DNA substitution using the program GARLI. −InL = 45468.255.

Since the monophyly of the *Polyommatus* section had not been previously tested and Bálint & Johnson's [10] hypothesis proposes that some of the Neotropical taxa belong to other sections, we first performed a tribal-level analysis including 50 taxa representing 29 of the 30 sections of Polyommatini (electronic supplementary material, table S1). Phylogenies were inferred using molecular characters from 4939 bp derived from six markers for each representative—two mitochondrial (*COI*–(*leu-tRNA*)–*COII*) and four nuclear markers (*EF-1*α**, *28S*, *H3* and *wg*). Although some relationships between sections were supported only in the BI analysis, the trees obtained using different methods—BI, ML and MP—showed strong agreement in their topology (figure 2; electronic supplementary material, figure S2 and table S6). All the sections for which more than one representative had been included in the study were recovered as monophyletic with good support, including the *Polyommatus* section. All combined analyses recovered and supported the *Everes* section as sister to the *Polyommatus* section, and *Leptotes* section as sister to both. Statistics, number of best trees and scores, as well as support values for the relevant nodes obtained for each method, are described in the electronic supplementary material.

**Figure 3.**
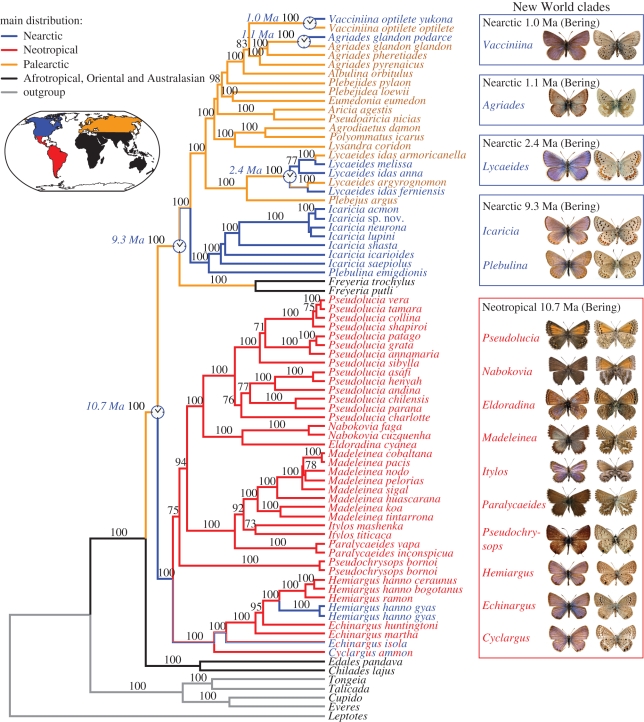
Phylogeny of the section *Polyommatus* (Lycaenidae: Polyommatini). Bayesian chronogram inferred from 6017 bp of two mitochondrial and six nuclear markers (*COI*–(*leu-tRNA*)–*COII*, *EF-1*α**, *28S*, *H3*, *ITS-2*, *CAD* and *wg*) for 73 taxa representing all New World genera and subgenera, their closest Old World relatives, and representatives of all Old World genera (*sensu* [29]). Colours indicate the main distribution of taxa and lineages according to ML ancestral area reconstruction analysis using Lagrange. Estimated ages for the five New World colonization events through Beringia are indicated at the corresponding nodes. Posterior probability values above 70 per cent are presented above recovered branches. Boxes to the right include the main distribution, age of colonization and route for each New World lineage, as well as photographs showing upperside and underside of a representative adult male specimen for each genus.

We then examined relationships within the *Polyommatus* section using 73 taxa representing all New World genera and subgenera, and all Old World taxa that had been hypothesized to be related to them (electronic supplementary material, table S1). The trees were inferred from 6017 bp fragments of eight markers: *COI*–(*leu*-*tRNA*)–*COII*, *EF-1*α**, *28S*, *H3*, *wg*, *CAD* and *ITS-2*. The trees generated using different methods (BI, ML and MP) shared the same basic topology (figure 3 and electronic supplementary material, table S6), and most of the relationships within the section were well resolved and supported. The Palaeotropical *Chilades* group (including *Edales*) was sister to the rest of the section. The Neotropical taxa formed a strongly supported monophyletic clade that was sister to the Holarctic taxa plus *Freyeria*. Systematic results are described and discussed in the electronic supplementary material.

Ancestral area reconstruction analysis using the program Lagrange [22] showed that at least five New World colonization events occurred, and that Beringia was the most likely route followed in all cases (electronic supplementary material, figure S6). The analysis using DIVA v. 1.1 [21] was compatible with this scenario, although less conclusive because it resulted in more than one equally parsimonious possibility for some nodes (electronic supplementary material, figure S5). Dating based on several published rates of evolution and methods of estimation resulted in the following mean ages for the five colonization events: 10.7 Ma for the Neotropical clade, 9.3 Ma for the *Icaricia*–*Plebulina* clade (which included the taxon *saepiolus*), 2.4 Ma for the *Lycaeides* clade, 1.1 Ma for *Agriades* and 1.0 Ma for *Vacciniina* (figures 1,3 and electronic supplementary material, table S7).

In order to test the feasibility that the colonizing ancestors could survive in Beringia at the times estimated, we reconstructed their palaeoecology. Ancestral character reconstruction based on current hostplant data unambiguously showed that Fabaceae was the most likely hostplant family for the three first colonizing ancestors, while Primulaceae and Ericaceae were the most likely hostplants for the ancestors of New World *Agriades* and *Vacciniina*, respectively (electronic supplementary material, figure S7). To study ancestral temperature tolerances, we first selected the coldest and warmest locations for each taxon in the phylogeny and used WorldClim v. 1.4 [24] to obtain the climatic data (electronic supplementary material, table S5). We then coded mean annual temperatures at coldest and warmest locations as two ordered continuous characters, and performed Markov chain Monte Carlo ancestral reconstructions using the program BayesTraits beta v. 1.1 [25]. The results showed covariation between the coldest and warmest mean temperatures tolerated by the taxa. Interestingly, the better fit of model B (directional) over model A (random walk) demonstrates directionality in the evolution of temperature tolerance. The estimated value for the scaling parameter *lambda* was close to 1 (0.925), which indicates that the evolution of thermal tolerances has a strong phylogenetic signal. Since estimating *delta* and *kappa* scaling parameters did not significantly affect the fit of the model to the data, the tempo of evolution (branch lengths and overall path lengths) of the tree agrees well with the thermal tolerance data. The reconstruction analysis shows that the ancestors of the more recent colonization events are more cold-adapted, and this trend is significant for the coldest temperatures tolerated (*p* < 0.01). The range of temperature tolerance of each New World colonizer matches the palaeoclimate in Beringia at the time of colonization (figure 1*b* and electronic supplementary material, table S8).

## 4. Discussion

Our molecular phylogeny indicates that the New World *Polyommatus* blues are polyphyletic, and constitute five separate invasions: (i) the origin of the Neotropical clade from Southeast Asian ancestors, which we estimate occurred around 10.7 Ma; (ii) the subsequent evolution in North America of the *Icaricia*–*Plebulina* clade (including the taxon *saepiolus*) around 9.3 Ma; (iii) the evolution of *Lycaeides* and its relatives around 2.4 Ma; (iv) the evolution of *Agriades* around 1.1 Ma; and finally (v) the evolution of *Vacciniina* around 1.0 Ma (figures 1,3 and electronic supplementary material, table S7). Our results show that Nabokov's inferences based on morphological characters (primarily of the male genitalia) were uncannily correct in delineating not only species relationships but also the historical ordering of these five key events in the evolution of New World blues.

In our tribal-level analysis, the Palaeotropical *Chilades* group is sister to the rest of the section *Polyommatus*. This supports the origin of the *Polyommatus* section in Southeast Asia, as reflected, too, by the great diversity of taxa in the section from this region [1]. The fact that the Neotropical taxa form a strongly supported monophyletic group that is not directly related to the Nearctic taxa refutes Bálint & Johnson's hypothesis [29] that the Neotropical taxa are polyphyletic in origin. Despite the uncertainty involved in any estimate based on a molecular clock, the estimated date of 10.7 Ma for the divergence of the Neotropical clade from Old World ancestors makes it impossible to invoke a Gondwanan origin for this group, which would have required an estimated date of divergence at least as early as 80 Ma.

Ancestral area reconstruction analyses, especially model-based ML inferences, indicate that Beringia is the most likely route followed in the five New World colonization events (electronic supplementary material, figures S5 and S6). The first involves a long journey by the ancestor of the Neotropical group. This lineage crossed Beringia around 10.7 Ma, according to our molecular clock estimates, and dispersed from the Western Nearctic to Central America, where it then radiated in all the Neotropics. No explicit trace of this history remains today, as the ancestors subsequently went extinct in North America, with the possible exception of the lineage that gave rise to *Echinargus isola*. Two species of this group secondarily colonized the southern Nearctic region from the south (*Hemiargus hanno* and *Cyclargus ammon*).

The other four lineages that subsequently reached the New World also followed the Beringia route, but did not extend as far south as the Neotropics, and eastwards only as far as the East Nearctic. This result agrees with the fact that the Northern Atlantic landbridge had long disappeared when the *Polyommatus* blues colonized the New World. However, our results demonstrate that taxa of the *Polyommatus* section crossed from Asia to Alaska before, but also well after, the formation of the Bering Strait [30], showing that the presence of a continuous landbridge was not a necessary requirement for the dispersal of these butterflies. A transoceanic dispersal event from Asia to the Neotropics cannot be completely ruled out for the Neotropical clade, although the likelihood of such a long-distance one-step colonization is low.

Ancestral temperature tolerance reconstruction analysis (figure 1*b* and electronic supplementary material, figure S8) shows that lineages involved in more recent colonization events across Beringia had ancestors that were more tolerant to the cold. Thus, the ancestors of the Neotropical stock, the least able to cope with cold conditions, were pushed southwards by decreasing temperatures and periods of glaciation during the post-Miocene [27,28,31], practically disappearing from North America. In contrast, the cold-adapted lineages could persist and even radiate in the north.

In a suggestive parallel with the Neotropical blues, some groups of extant Neotropical Fabaceae—the hostplant family of the colonizing ancestors, according to our character reconstruction analysis (electronic supplementary material, figure S7)—seem to represent Tertiary boreotropical relicts that were previously widely distributed in North America [32]. Moreover, *Trifolium* and *Astragalus*, arguably the most widely used hostplant genera by New World *Polyommatus* butterflies [33], also appear to have originated in Eurasia and colonized North and South America within the last 10 Myr, although in these cases the exact routes are not known [34–36]. Thus, the palaeoecology of *Polyommatus* blues, estimated using both hostplant and temperature requirements, further supports the area reconstruction and dating results for this clade.

The fact that the time of colonization is significantly correlated with temperature tolerances of colonizing taxa indicates that while climate change has not stopped dispersal across this route, it has strongly filtered migrants according to their thermal tolerances. Other species of butterflies could have followed this same route [37–39], as well as other animal and plant groups [40–44]. Our data indicate that Beringia served as a gateway between the Old and the New World that was regulated by climate change, both for warm-adapted organisms during the Miocene and later for cold-adapted ones, including humans, able to cross the strait. The match between coldest temperatures tolerated by the butterfly ancestors and the palaeoclimate in Beringia (figure 1) provides independent support for the Miocene–Pleistocene climate change based on phylogeny and present-day ecological requirements. These findings indicate substantial phylogenetic niche conservatism (defined as retention of ecological traits over time among related species), not only for hostplant preferences as generally accepted [45,46], but also for thermal tolerances. The strong phylogenetic signal in thermal tolerances is also indicated by the estimated value of the *lambda* scaling parameter (0.925), which is close to one. Nevertheless, evolution in this character exists because extant taxa do differ in their temperature ranges (electronic supplementary material, figure S8). Conservatism in the adaptation to specific climates has been documented in few instances [47–49], and is probably more marked in mobile organisms that can disperse to suitable habitats as conditions change, thus reducing selective pressure for adaptation. Evidence for conservatism in thermal tolerances is of importance because it is an intrinsic assumption underlying the application of ecological niche modelling to reconstruct ancestral geographical ranges and predict future species distributions according to climate change scenarios [50–54].

## 5. Conclusions

— The phylogeny of the tribe Polyommatini (Lycaenidae) is inferred for 50 taxa representing 28 of the 29 sections (*sensu* [1], excluding *Cupidopsis*) using 4939 bp from six markers (two mitochondrial, four nuclear) for each representative. The *Polyommatus* section is recovered as monophyletic and sister to the *Everes* section, with both sister to the *Leptotes* section.
— The phylogeny of the section *Polyommatus* (Lycaenidae: Polyomatini) is inferred for 73 taxa representing all New World genera and subgenera, their closest Old World relatives, and representatives of all Old World genera (*sensu* [29]) using 6017 bp from eight markers for each representative (two mitochondrial and six nuclear).
— Phylogenetic results support Vladimir Nabokov's [8] hypothesis that the New World *Polyommatus* are the product of at least five colonization events through Beringia that occurred successively from *ca* 11 Ma until 1 Ma. Although Beringia was still intact during the earliest of these crossings, later colonizers did not require a continuous landbridge.
— Fabaceae was the most likely hostplant family for the three first colonizing ancestors, while Primulaceae and Ericaceae were the most likely hostplant families for the ancestors of New World *Agriades* and *Vacciniina*, respectively. These hostplant groups are thought to have existed in the New World before the arrival of *Polyommatus* butterflies, or to have colonized concurrently with them.
— A novel method is used to estimate ancestral temperature tolerances using the limits of distribution ranges of extant organisms.
— Each of the five colonizing ancestors was thermally adapted to Beringian palaeoclimatic conditions at the time of its transcontinental dispersal, with earlier colonists being more warm-adapted than later ones in accordance with changing temperatures in Beringia. *Polyommatus* butterflies show substantial phylogenetic conservatism in thermal tolerances, although evolution in this trait is also observed.
— Climate fluctuations in Beringia thus acted as a filter that influenced which groups of *Polyommatus* butterflies could colonize the New World. These results demonstrate the importance of climate change in governing biogeographic patterns as well as the extent of Nabokov's extraordinary biological intuition.
